# Supplementary material for: Incorporating human mobility data improves forecasts of Dengue fever in Thailand
Source: Sci Rep. 2021 Jan 13;11:923. doi: 10.1038/s41598-020-79438-0 (PMC7806770; doi:10.1038/s41598-020-79438-0)
Supplement: Supplementary file 1 — Supplementary Information 1. [file 41598_2020_79438_MOESM1_ESM.docx]

**SI Appendix: Incorporating human mobility data improves forecasts of
Dengue fever in Thailand**

Mathew V Kiang ScD^a,$^, Mauricio Santillana PhD^b,c,$^, Jarvis T Chen ScD^d^, Jukka-Pekka Onnela DSc^e^, Nancy Krieger PhD^d^, Kenth Engø-Monsen PhD^f^, Nattwut Ekapirat MSc^g^, Darin Areechokchai MD MCTM^h^, Preecha Prempree MD PhD^h^, Richard J. Maude MD DPhil,^g,i,k,^*^†^* and Caroline O Buckee PhD^j,k,1,^*^†^*


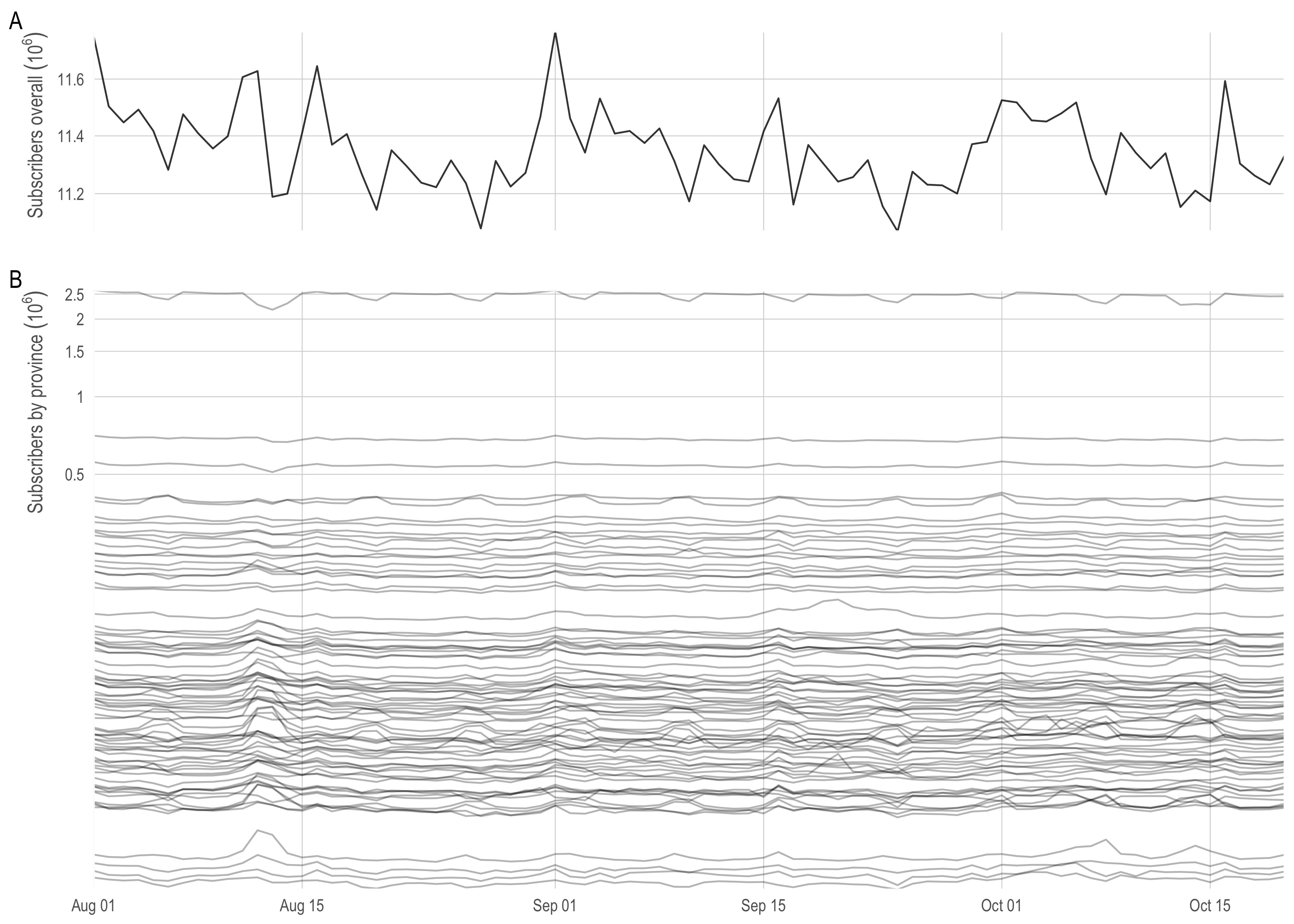


**Figure S1. Number of daily cellular subscribers nationally (top) and by province (bottom).** The number of daily cellular subscribers (in millions) remained stable over the period of observation with a coefficient of variation of 1.3% nationally.

*
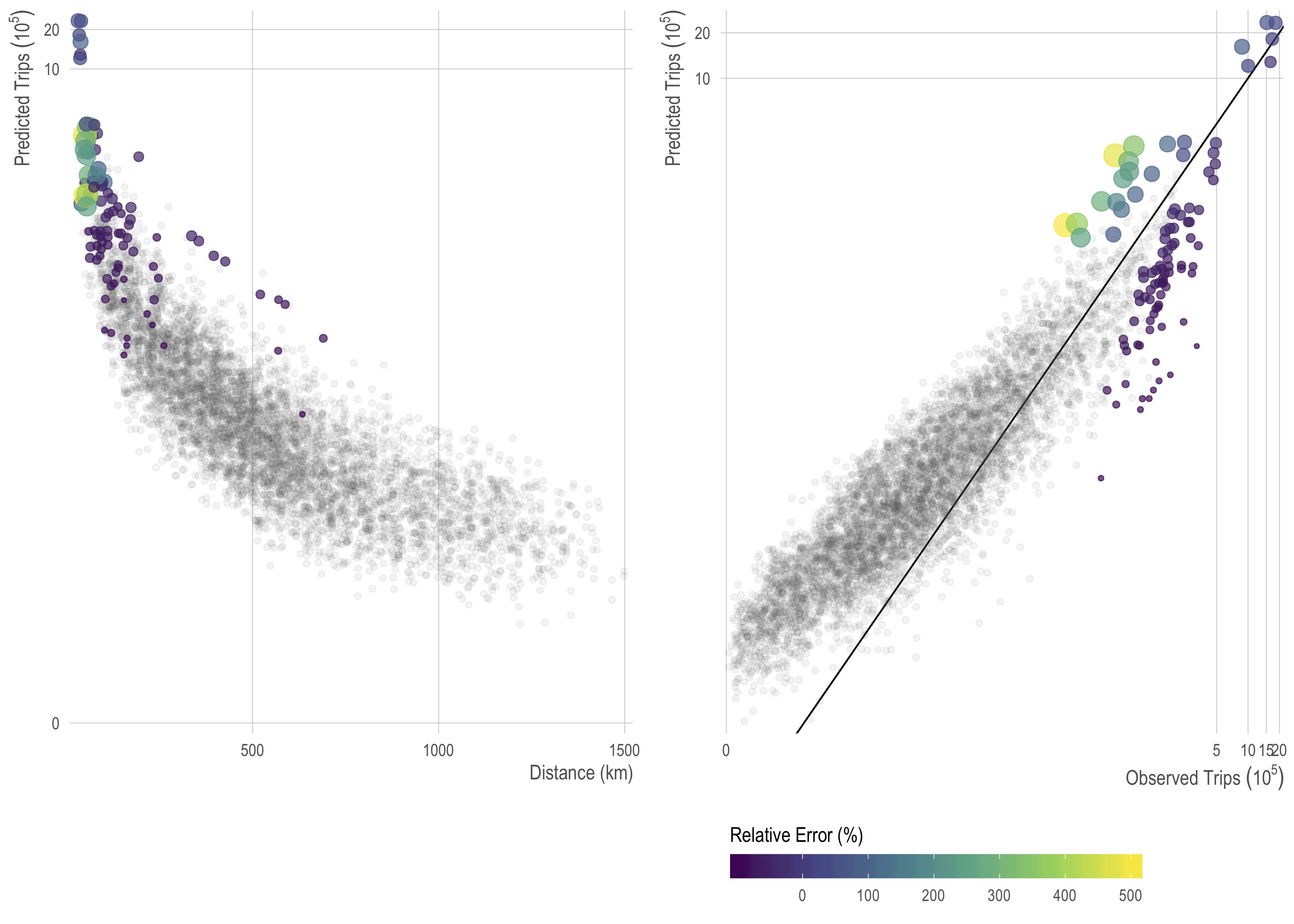
*

**Figure S2. Outlier over- and under-prediction of mobility.** We show observed number of trips in the mobile phone data (y-axis) over distance (left panel; x-axis) and compared to the number of trips predicted by the gravity model (right panel; x-axis). In both panels, we highlight outlier observations (defined as observations with a Cook’s distance greater than 5 times the mean Cook’s distance) with the size and color of the relative error defined as 100%*(PredictedTrips – ObservedTrips)/ObservedTrips. We note that that the outliers have both under- and over-prediction. See Figure 1 to see these outliers plotted on a map.


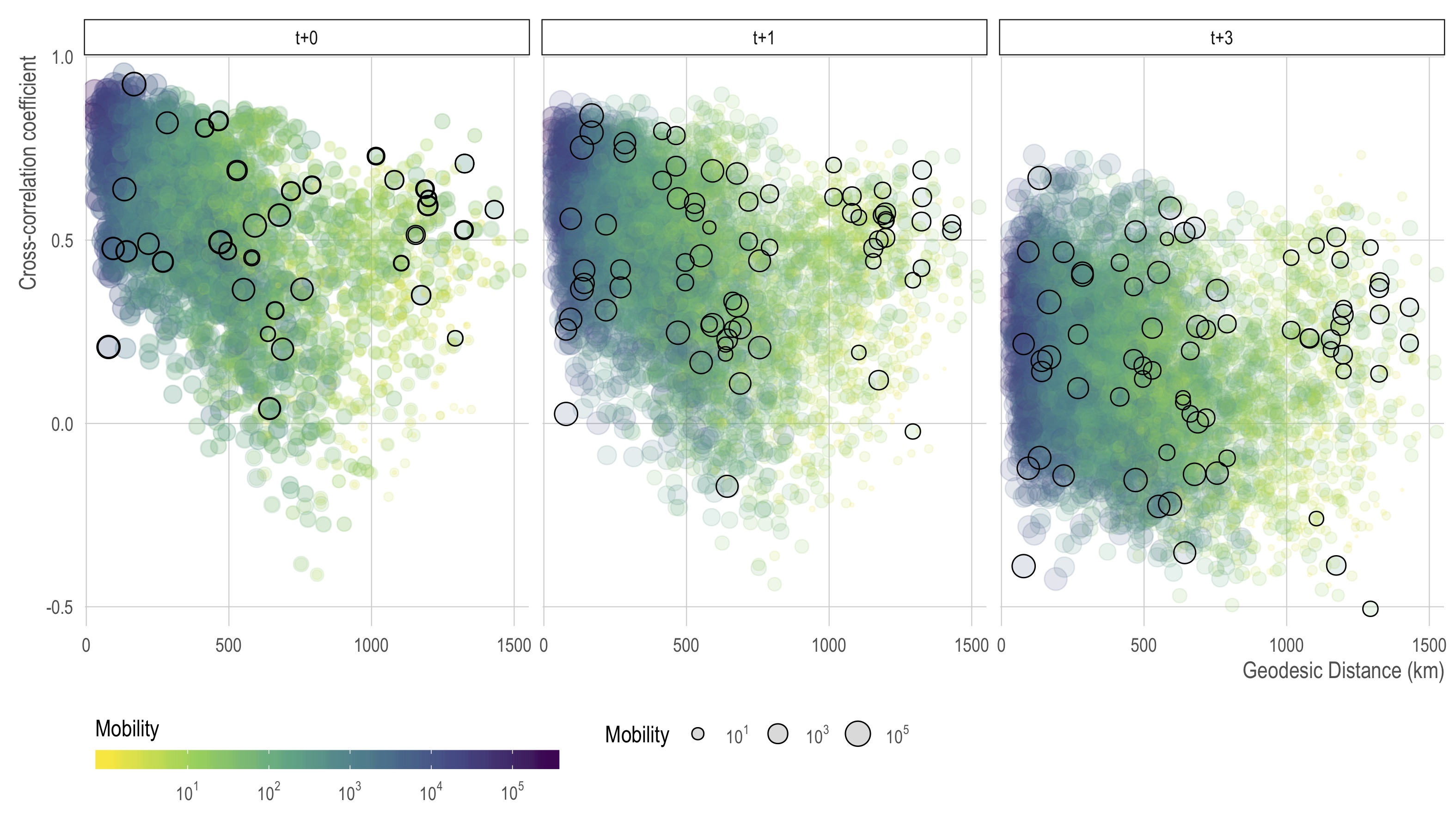


**Figure S3. Cross-correlation of dengue incidence by distance.** For each pair of provinces, we calculated the cross-correlation with lags of 0, 1, and 3 months. We cross-correlation coefficient (y-axis) against the distance between pairs (x-axis). Consistent with previous research, there appears to be lower correlation over longer distances in general, and this effect is exacerbated with higher time lags. We outline in black observations between two provinces that each have an international airport and note that many long-distance, high-correlation observations are between these provinces — see Figure S4.

**Figure S4. Comparing cross-correlation coefficients of provinces connected by airports to those not connected by airports.** For each forecasting horizon between t+1 and t+6 months, we compare the empirical cumulative distribution function of the cross-correlation coefficients provinces connected by airports (blue) to those not connected by airports (red). In the upper left of each panel, we show the Kolmogorov-Smirnov test statistic (*D*) and associated p-value. In farther forecasting horizons (i.e., 3-6 months in advance), provinces connected by airports are more correlated than those not connected by airports.


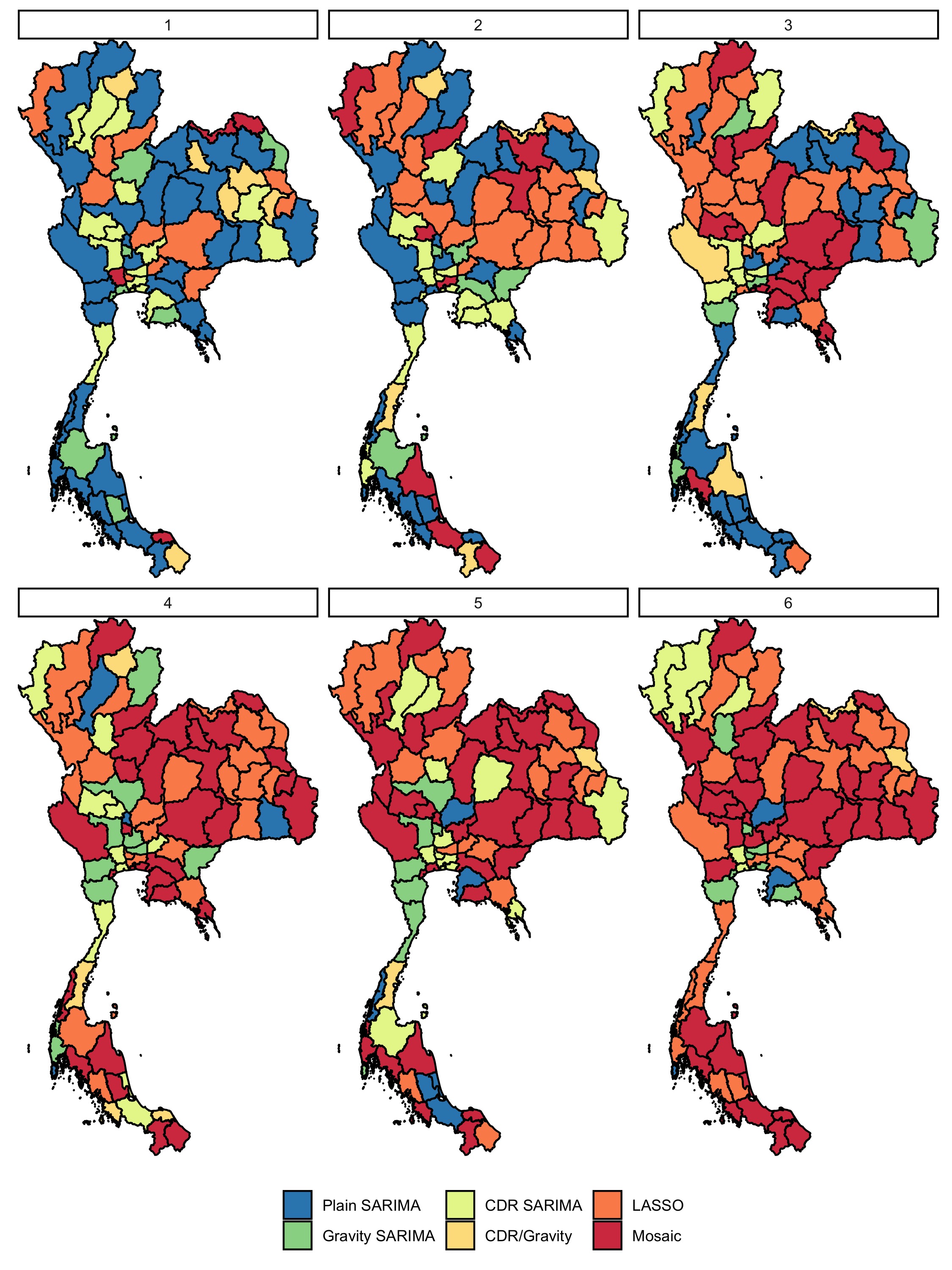


**Figure S5. Class of the best performing model for each province and forecasting horizon.** For each forecasting horizon between t+1 and t+6 months, we plot the class of the best performing model (i.e., lowest mean absolute error). In farther forecasting horizons, the LASSO and mosaic models outperform SARIMA-based models. Note: in some instances, both the CDR and Gravity SARIMAs are equivalent. All plots were made using ggplot2^1^ in R 4.0.1^2^.

**Figure S6. Assessing the performance of models with mobile phone data to those without mobile phone data.** For each forecasting horizon between t+1 and t+6 months and region, we compare the out-of-sample prediction errors of the best performing CDR SARIMA to the best performing model of other classes. Models were compared using a Wilcoxon signed-rank test. Statistically significantly different models are shown with red dots indicating better CDR SARIMA performance relative to the comparison model and blue dots indicating worse performance.

**Figure S7. Assessing the performance of the mosaic model to an AR(1).** For each forecasting horizon between t+1 and t+6 months and region, we compare the out-of-sample prediction errors of the mosaic model to a naïve AR(1) model. Models were compared using a Wilcoxon signed-rank test. Statistically significantly different models are shown with red dots indicating better performance of the mosaic model relative to the AR(1) model and blue dots indicating worse performance.


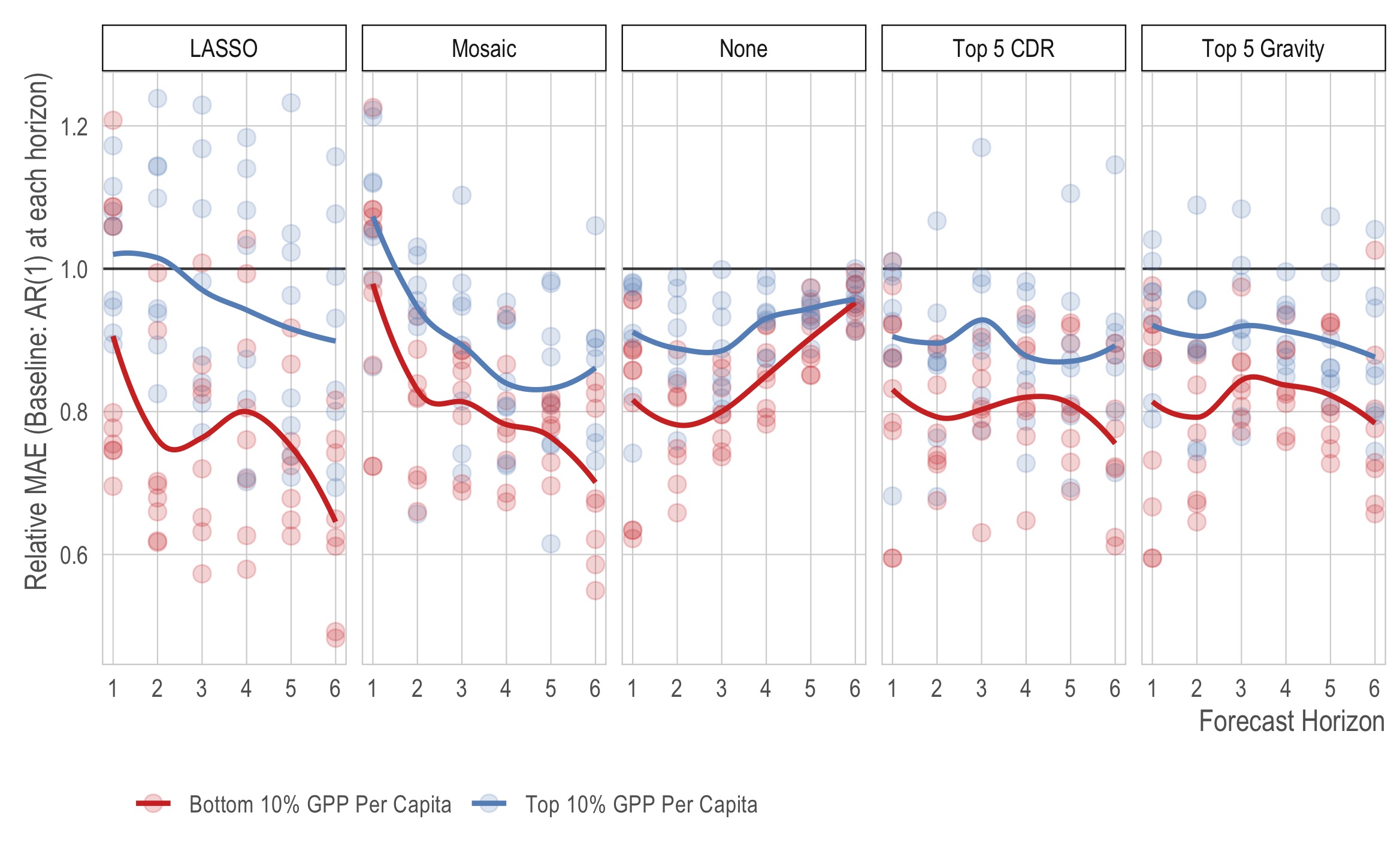


**Figure S8. Comparing the model performance of areas with high and low gross provincial product (GPP) per capita at different forecasting horizons.** For each forecasting horizon between t+1 and t+6 months and model class, we compare the best model for the top (blue) and bottom (red) 10% areas in terms of gross provincial product per capita. The y-axis is the relative mean absolute error (MAE) compared to a traditional AR(1) autoregressive model. Lower numbers indicate better predictive accuracy.


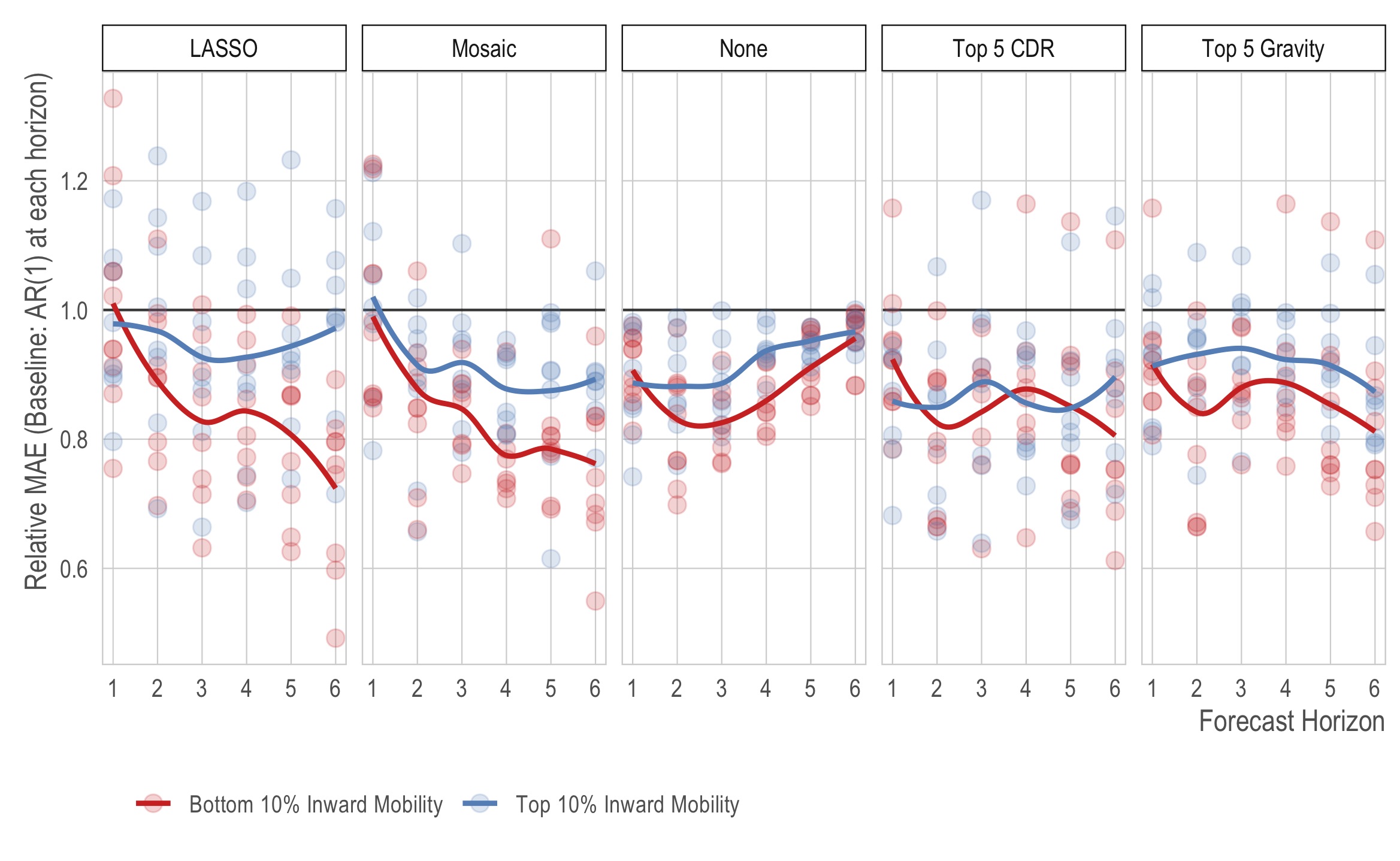


**Figure S9. Comparing the model performance of areas with high and low inward mobility at different forecasting horizons.** For each forecasting horizon between t+1 and t+6 months and model class, we compare the best model for the top (blue) and bottom (red) 10% areas in terms of inward mobility. The y-axis is the relative mean absolute error (MAE) compared to a traditional AR(1) autoregressive model. Lower numbers indicate better predictive accuracy.


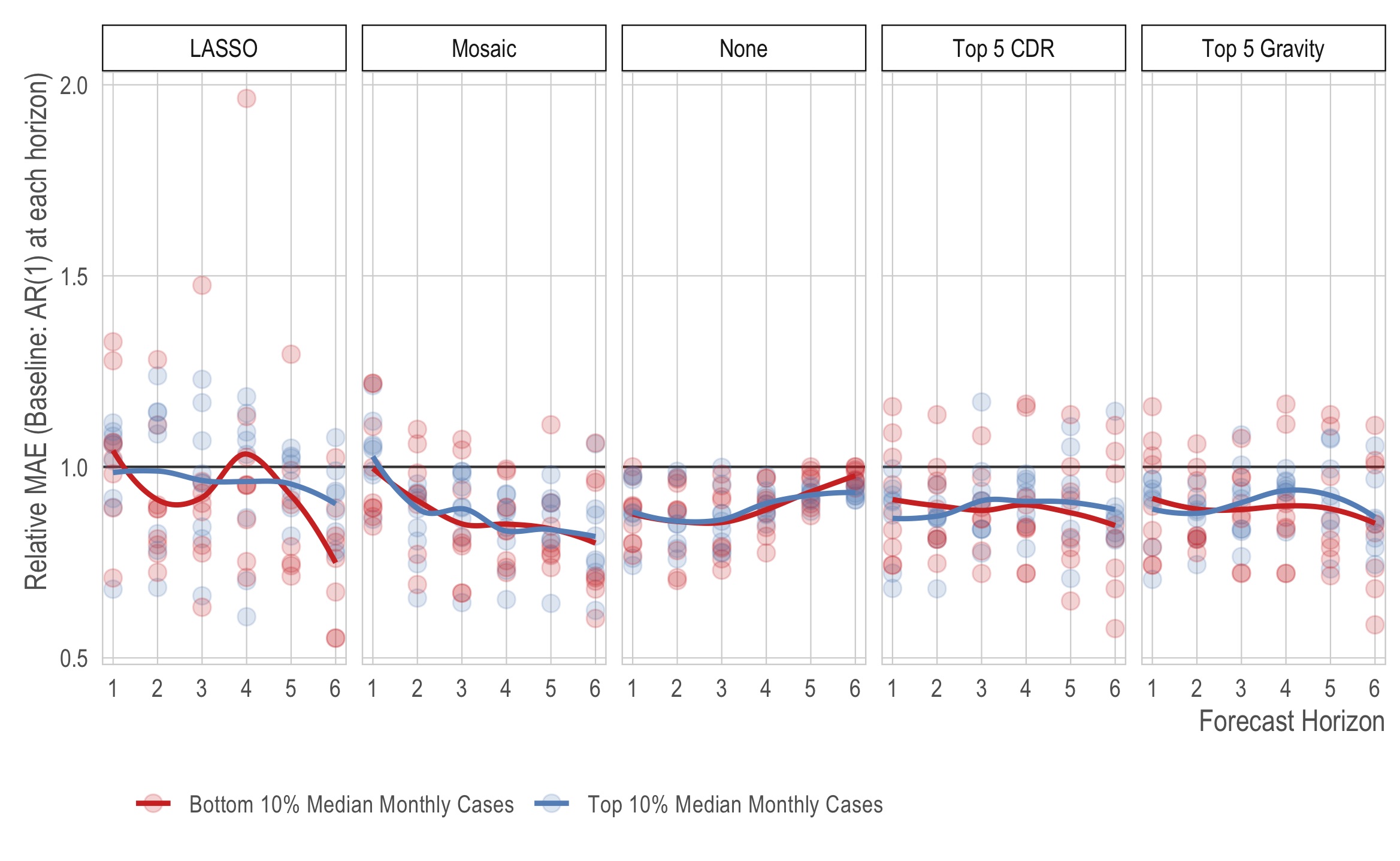


**Figure S10. Comparing the model performance of areas with high and median monthly dengue cases at different forecasting horizons.** For each forecasting horizon between t+1 and t+6 months and model class, we compare the best model for the top (blue) and bottom (red) 10% areas in terms of median monthly dengue cases. The y-axis is the relative mean absolute error (MAE) compared to a traditional AR(1) autoregressive model. Lower numbers indicate better predictive accuracy.


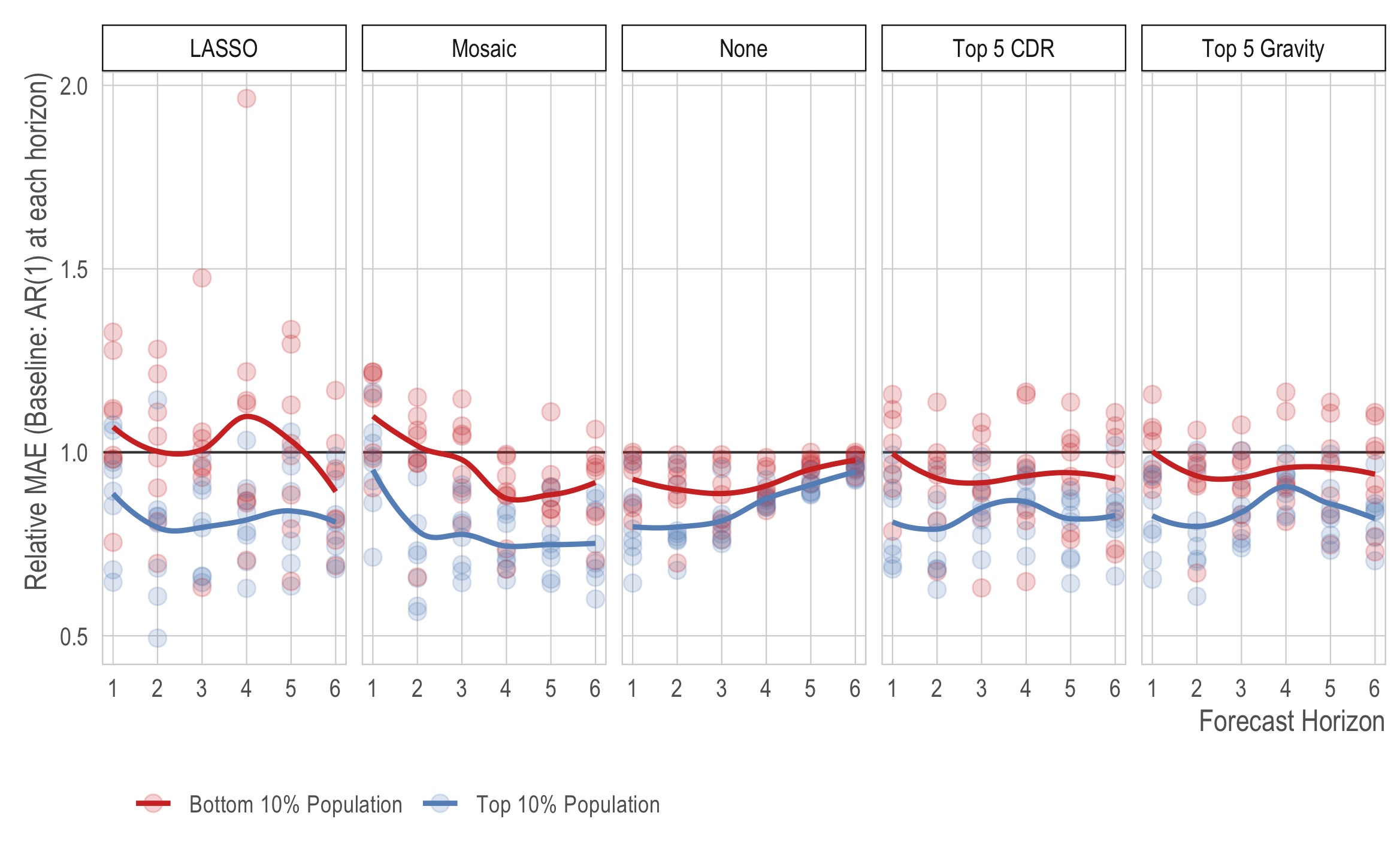


**Figure S11. Comparing the model performance of areas with high and low population at different forecasting horizons.** For each forecasting horizon between t+1 and t+6 months and model class, we compare the best model for the top (blue) and bottom (red) 10% areas in terms of population. The y-axis is the relative mean absolute error (MAE) compared to a traditional AR(1) autoregressive model. Lower numbers indicate better predictive accuracy.


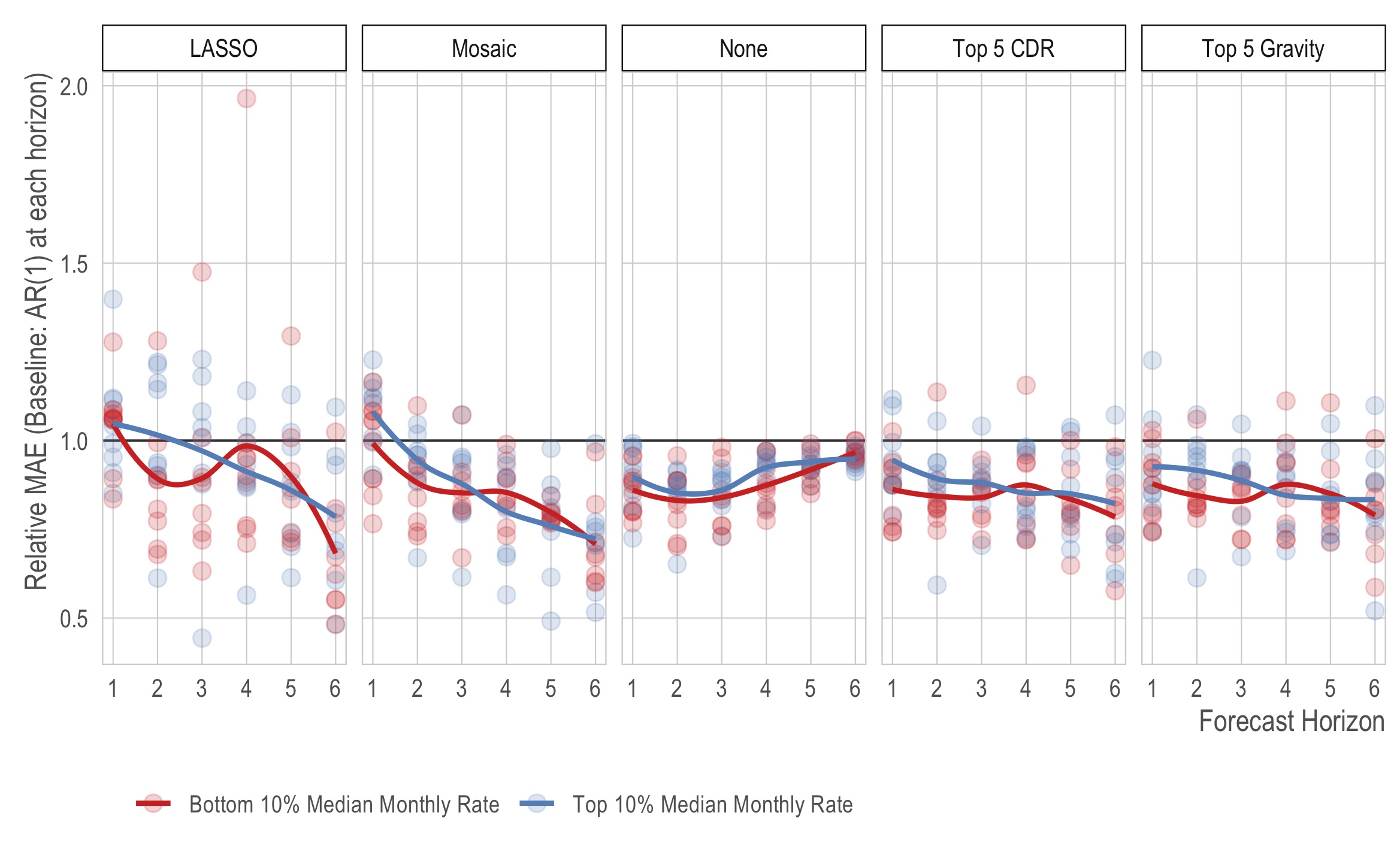


**Figure S12. Comparing the model performance of areas with high and low median monthly crude dengue incidence rate at different forecasting horizons.** For each forecasting horizon between t+1 and t+6 months and model class, we compare the best model for the top (blue) and bottom (red) 10% areas in terms of the median monthly dengue incidence rate. The y-axis is the relative mean absolute error (MAE) compared to a traditional AR(1) autoregressive model. Lower numbers indicate better predictive accuracy.


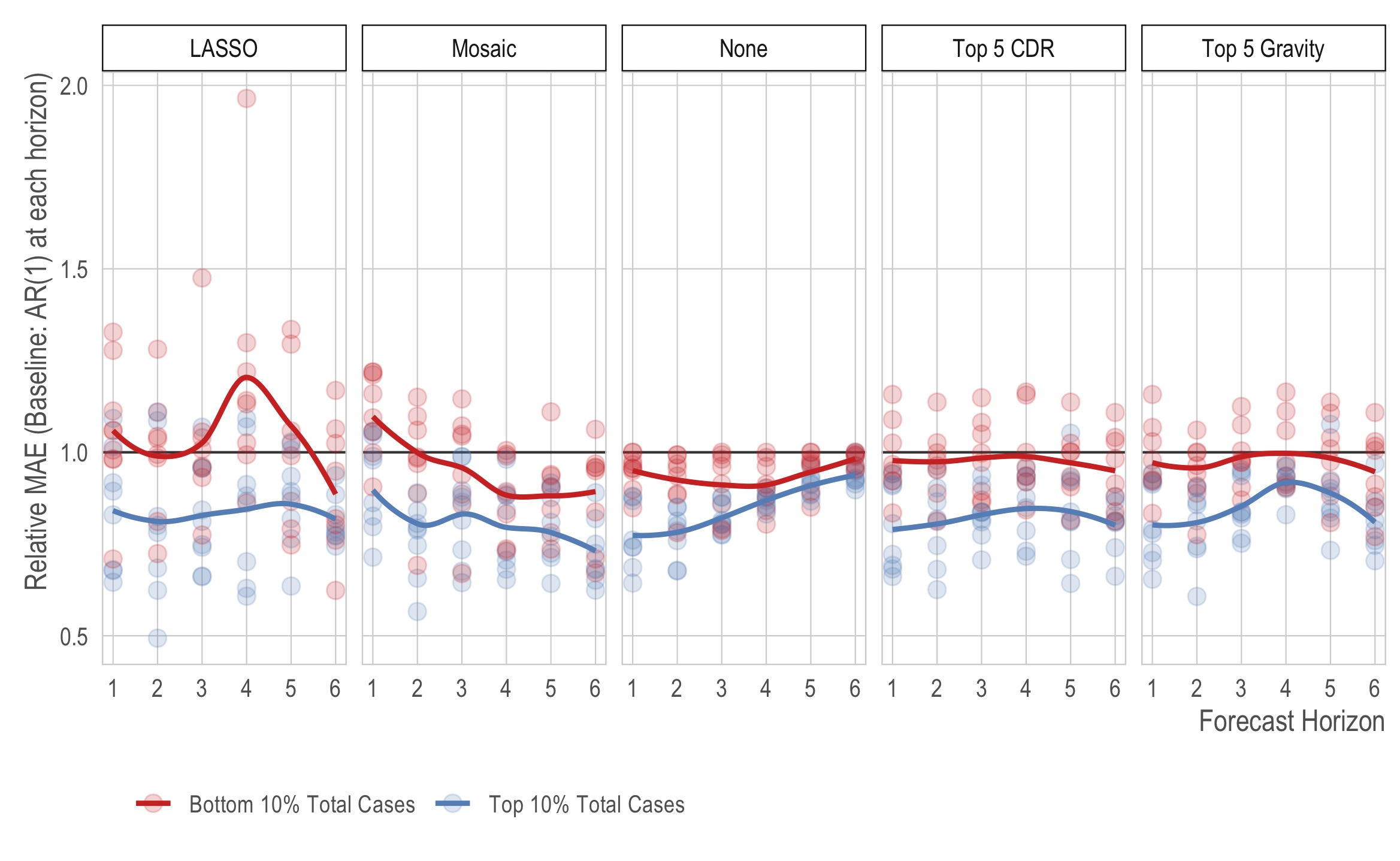


**Figure S13. Comparing the model performance of areas with high and low total dengue cases at different forecasting horizons.** For each forecasting horizon between t+1 and t+6 months and model class, we compare the best model for the top (blue) and bottom (red) 10% areas in terms of total dengue cases. The y-axis is the relative mean absolute error (MAE) compared to a traditional AR(1) autoregressive model. Lower numbers indicate better predictive accuracy.

**Table S1. Gravity model results.** The estimates and standard errors of each parameter for the gravity models by three distance measures: geodesic distance, road distance, and travel time. We present both unadjusted model estimates and after adjusting for gross provincial product (GPP) per capita. To assess best fit, we used normalized root mean squared error (NRMSE).

|  | **Geodesic Distance** | | **Road Distance** | | **Travel Time** | |
| --- | --- | --- | --- | --- | --- | --- |
|  | Unadjusted | Adjusted | Unadjusted | Adjusted | Unadjusted | Adjusted |
| Constant ($k$) | –3.795 (0.319) | –2.696 (0.320) | –2.162 (0.347) | –0.822 (0.353) | –1.201 (0.391) | –1.527 (0.409) |
| Population *i* ($\alpha$) | 0.632 (0.014) | 0.767 (0.015) | 0.602 (0.015) | 0.744 (0.016) | 0.587 (0.016) | 0.751 (0.018) |
| Population *j* ($\beta$) | 0.785 (0.013) | 0.716 (0.014) | 0.743 (0.014) | 0.672 (0.015) | 0.722 (0.015) | 0.683 (0.016) |
| Distance *i* to *j* ($\gamma$) | -1.624 (0.014) | -1.693 (0.015) | -1.653 (0.015) | -1.787 (0.018) | -1.787 (0.018) | -1.947 (0.020) |
| GPP per capita *i* ($\psi$) |  | –0.451 (0.022) |  | –0.468 (0.023) |  | –0.557 (0.025) |
| GPP per capita *j* ($\phi$) |  | 0.250 (0.019) |  | 0.239 (0.020) |  | –1.947 (0.020) |
|  |  |  |  |  |  |  |
| Observations | 5,847 | 5,847 | 5,847 | 5,847 | 5,847 | 5,847 |
| NRMSE | 0.018 | 0.017 | 0.016 | 0.016 | 0.014 | 0.013 |
| Pseudo-R^2^ | 0.859 | 0.873 | 0.853 | 0.868 | 0.838 | 0.858 |

**Text S1. Province-specific reports**. In an online-only supplement, we have provided detailed model results for each province separately. These results include the most connected provinces by both observed CDR data and estimated gravity models as well as the results for each model. These files can be viewed at: <https://osf.io/m6gnr/>.

**Text S2. Transition probabilities**. In an online-only supplement, we have provided an aggregated, de-identified probability transition matrix for province-to-province travel. For pair of provinces (*i*, *j*), we provide the population-weighted mean probability of travel from *i* to *j* (i.e., travel from *i* sums to 1). These data can be accessed as a csv file in long format or as a matrix object in *R*. These files can be viewed at: <https://osf.io/m6gnr/>.

**Supplemental References**

1. Wickham, H. *ggplot2: Elegant Graphics for Data Analysis*. (Springer-Verlag New York, 2016).

2. Team, R. C. R: A Language and Environment for Statistical Computing. *R Foundation for Statistical Computing* (2018).
